# The Casual Associations Between Brain Functional Networks and Fibromyalgia: A Large-Scale Genetic Correlation and Mendelian Randomization Study

**DOI:** 10.3390/bioengineering12070692

**Published:** 2025-06-25

**Authors:** Yiqun Hu, Guang Yang, Zhenhan Deng, Shengwu Yang, Yusheng Li, Wenfeng Xiao, Bangbao Lu, Xiongbai Zhu

**Affiliations:** 1 Department of Orthopedics, Xiangya Hospital, Central South University, Changsha 410000, China; 8303211705@csu.edu.cn (Y.H.); 228111092@csu.edu.cn (G.Y.); liyusheng@csu.edu.cn (Y.L.); xiaowenfeng@csu.edu.cn (W.X.); 2 Department of Orthopedics, The First Affiliated Hospital of Wenzhou Medical University, Wenzhou 325000, China; dengzhenhan@wmu.edu.cn (Z.D.); yangshengwu188@sina.com (S.Y.); 3 Geriatrics Center, The First Affiliated Hospital of Wenzhou Medical University, Wenzhou 325000, China; 4 National Clinical Research Center for Geriatric Disorders, Department of Geriatrics, Xiangya Hospital, Central South University, Changsha 410000, China

**Keywords:** fibromyalgia, brain functional networks, resting-state functional MRI, mendelian randomization, genetic correlations

## Abstract

While the central mechanisms of fibromyalgia have gained attention, the causal effects between brain networks and fibromyalgia remain unclear. Two-sample Mendelian randomization and Linkage Disequilibrium Score Regression were performed to investigate the relationship between 191 rsfMRI traits and 8 fibromyalgia-related traits. A total of 4 rsfMRI traits were genetically correlated with trouble falling asleep, 11 with back pain for 3+ months, 16 with pain all over the body, 14 with insomnia, 5 with fibromyalgia, 4 with fibromyalgia, and 3 with malaise and fatigue. Pheno801 has significant causal effects on malaise and fatigue (OR = 1.0022, *p* = 0.01), fibromyalgia (finngen) (OR = 1.5055, *p* = 0.03), and insomnia (OR = 1.4063, *p* = 0.04). Pheno1696 significantly impacts fibromyalgia-related comorbidities (OR = 1.002, *p* = 0.02), trouble falling asleep (OR = 1.0285, *p* = 0.04), malaise and fatigue (OR = 1.0011, *p* = 0.04), and pain all over the body (OR = 0.9967, *p* = 0.04). Pheno103 has marked effects on fibromyalgia (finngen) (OR = 0.7477, *p* = 0.02), malaise and fatigue (OR = 0.9987, *p* = 0.03), and pain all over the body (OR = 1.0033, *p* = 0.03). Our findings suggest that targeting these networks could effectively prevent or alleviate fibromyalgia.

## 1. Introduction

Fibromyalgia, or fibromyalgia syndrome, represents a leading cause of chronic widespread pain (CWP), primarily characterized by pain and accompanied by a complex array of symptoms including fatigue, sleep disturbances, and various functional impairments [1]. It ranks as the third most prevalent musculoskeletal disorder, following lumbar pain and osteoarthritis [2]. The prevalence of this condition tends to increase with age, reaching its peak between the ages of 50 and 60 [3], with a global average incidence rate of approximately three women to every man [4]. The quality of life for those suffering from fibromyalgia is generally poor, as reflected by significant health-care expenditures due to frequent medical consultations [1]. Patients with fibromyalgia typically require nearly twice the number of medical consultations compared to healthy individuals [5], and their total health-care costs are estimated to be threefold higher than those of other individuals, based on comparisons with a random population sample [6]. Additionally, research by Guymer has shown that 24.3% of patients cease working within five years of the onset of fibromyalgia [7].

Currently, the pathogenesis of fibromyalgia lacks a consensus [1], yet there is growing recognition of the central mechanisms’ crucial role in this condition. Functional brain networks underlie the sophisticated functions of the human brain, and the connectivity and communication between these networks are fundamental to cognition, behavior, and emotion [8]. The human brain remains active during the resting state, and resting-state functional magnetic resonance imaging (rsfMRI) serves as a non-invasive method to visualize functionally active brain regions [8]. rsfMRI, a blood oxygen level-dependent (BOLD) magnetic resonance imaging technique, capitalizes on the magnetization differences between oxyhemoglobin and deoxyhemoglobin to generate the fMRI signal [9]. Studies, such as those by Cifre et al., reveal that patients with fibromyalgia demonstrate a significant imbalance in connectivity within the pain network during rest [10]. According to Napadow et al., these patients exhibit enhanced connectivity within the Default Mode Network (DMN) and right Executive Attention Network (EAN), as well as increased connectivity between the DMN and the insular cortex, a region known to process evoked pain [11]. Aoe et al. found that patients with a low pressure pain threshold show notably higher functional connectivity from the thalamus to the insular cortex and significantly lower connectivity between the secondary somatosensory area and the dorsal attention network [12]. These observational studies, predominantly cross-sectional or case-control in design, often face limitations in sample size due to research costs or ethical constraints, complicating the adjustment for confounding factors such as lifestyle, occupation, or education level between groups. Therefore, high-quality research designs are still required to confirm the causal relationships.

Mendelian randomization (MR) analysis is a statistical approach that infer potentially causal relationships from observational association results. By using genetic variants identified in genome-wide association study (GWAS) to construct instrumental variables, MR analysis can estimate the causal relationship between exposure and outcomes [13]. MR is an intermediate type between traditional epidemics and randomized controlled trials, and the level of evidence is somewhere in between. Compared to case-control and cohort studies, MR is characterized by a higher level of evidence [14]. Linkage Disequilibrium Score Regression (LDSC) is a well-established method utilized for the analysis of genetic correlation [15]. This approach effectively differentiates the inflation of statistical estimates attributed to polygenic genetic architecture from that resulting from population stratification or other confounding factors [16]. Furthermore, LDSC can be employed to estimate the genetic correlation between single nucleotide polymorphisms (SNPs) and various traits, enhancing our understanding of the genetic underpinnings of complex diseases [16].

In this study, we conducted a genetic correlation analysis using a GWAS dataset comprising rsfMRI traits and fibromyalgia-related traits. We initially employed LDSC to assess the heritability of the GWAS data. Subsequently, we estimated the causal effect sizes of rsfMRI traits on the risk of fibromyalgia using the MR method, with the robustness of our findings further validated through sensitivity analyses and LDSC. We also performed tests for heterogeneity and pleiotropy regarding our results. Our findings provide a foundation for future interventions aimed at targeting specific brain regions to normalize abnormal brain functional connectivity, thereby contributing to the treatment of fibromyalgia.

## 2. Materials and Methods

### 2.1. Data Source

#### 2.1.1. GWASs of Brain rsfMRI Traits

A total of 191 rsfMRI traits were extracted for our study from the research conducted by Zhao et al. [17]. Briefly described, a GWAS was performed on 1777 intrinsic brain activity traits alongside 9,026,427 common variants within the UKB British cohort, consisting of 34,691 participants [17]. It characterized 603 significant locus–trait associations across 191 traits, which include 75 amplitude traits, 111 pairwise functional connectivity, and 5 global functional connectivity traits, distributed over 45 genomic regions [17]. Detailed information about these datasets can be found in the original publication and is displayed in Table S1.

#### 2.1.2. GWASs of Fibromyalgia-Related Traits

Necessary GWAS data on fibromyalgia-related traits was retrieved from 3 sources: FinnGen [18], NealeLab [19], and the NHGRI-EBI GWAS Catalog [20]. Fibromyalgia was selected as the main outcome, along with 3 cardinal features of fibromyalgia as related outcomes, including sleep disturbances, pain, and fatigue [1]. The FinnGen study is a large-scale genomics initiative that has analyzed over 500,000 Finnish biobank samples and correlated genetic variation with health data to understand disease mechanisms and predispositions [21]. The project is a collaboration between research organizations and biobanks within Finland and international industry partners [21]. R11 data released on 24 June 2024 was obtained, which includes GWAS data related to Fibromyalgia (FinnGen) and insomnia. In 2018, Neale Lab released an updated (second) round of GWAS results from the UK Biobank, which may be freely downloaded and used without restriction [19]. GWAS data on pain all over the body, back pain for 3+ months, malaise and fatigue, fibromyalgia-related comorbidities, and trouble falling asleep was obtained from the Neale Lab. Summary statistics for Fibromyalgia (gcst) were downloaded from the NHGRI-EBI GWAS Catalog on 18 September 2024, for study GCST90129439 [22]. Detailed GWAS information is provided in Table S2.

### 2.2. Selection of Genetic Instruments

In the MR study, valid genetic instrumental variables (IVs) are required to meet 3 critical assumptions to ensure the integrity of causal inferences (8): (1) the IVs must be strongly associated with the exposures. (2) The IVs must be independent of the potential confounders of the association between the exposure and outcome; (3) the IVs should not be associated with the outcomes directly.

To meet the 3 critical assumptions and ensure the accuracy and authenticity of the MR results, the following quality control processes to select appropriate intravenous fluids were implemented. First, SNPs significantly associated with rsfMRI (*p* < 5 × 10^−6^) were chosen as instrumental variables (IVs). Second, due to linkage disequilibrium (LD) being a major source of bias in Mendelian randomization studies, it is crucial to eliminate genetically correlated variants to enhance the reliability of the results [23]. A clumping process (r^2^ > 0.001, clumping distance = 1000 kb) was employed to assess the LD among the included IVs. Third, the strength of the IVs was quantified using the proportion of variation explained (R2) and the F statistic. The F statistic is calculated as R2 = 2 × MAF × (1 − MAF) × β2, F = R2 (n − 2)/(1 − R2) (n = sample size; k = number of IVs) [24]. SNPs with F < 10 were deleted, for it indicated a weak instrumental variable bias. Fourth, SNPs with a minor allele frequency of < 0.01, ambiguous SNPs with non-concordant alleles, and palindromic SNPs were excluded. Fifth, Steiger analysis was used to confirm the causal direction [25]. Lastly, SNPs directly associated with fibromyalgia-related traits (*p* < 5 × 10^−8^) were excluded. Table S4 presents the final IVs included in our analysis.

### 2.3. Genetic Correlation Analysis

Linkage Disequilibrium Score Regression (LDSC) is a tool for analyzing genetic correlations, examining the association between test statistics and linkage disequilibrium to quantify the contribution of inflation from a true polygenic signal or bias [15]. This method can evaluate genetic correlation from GWAS summary statistics and is not biased by sample overlap [26]. Single-trait LD Score regression was utilized to calculate the overall heritability (h2), Genomic Inflation Factor (Lambda GC), intercept, and mean chi-squared for single GWAS summary data. A significant deviation of Lambda GC from one indicates the presence of population stratification in the GWAS summary data [16]. Additionally, LDSC was used to estimate the genetic correlation (rg) between rsfMRI traits and fibromyalgia-related traits as a supplementary method to assess their correlation.

### 2.4. Statistical Analysis of MR Study Design

In this study, 5 methods including inverse-variance weighted (IVW), weighted median, MR-Egger regression, weighted mode, and simple mode were used to examine the potential causal relationships between rsfMRI traits and fibromyalgia-related traits. The IVW results were primarily referenced for their ability to more precisely determine the impact of exposure on the outcomes when all selected SNPs are valid IVs [27,28]. In the discovery stage, MR analyses using SNPs significantly associated with rsfMRI (*p* < 5 × 10^−6^) as IVs were conducted to derive primary results. This primary estimate is then supported by additional analyses through complementary methods. Subsequently, we performed MR analyses in the replication stage, selecting instrumental variables with stronger associations with rsfMRI features (*p* < 5 × 10^−8^), and conducted additional MR analyses to further confirm the stability of our results.

To evaluate the heterogeneity among IVs, Cochran’s IVW Q statistics were utilized, with the choice of a random-effects IVW method if heterogeneity was detected (*p* < 0.05). Otherwise, a fixed-effects IVW approach was applied. For the investigation of horizontal pleiotropy effects, both the MR-Egger Intercept Test and the Mendelian Randomization Pleiotropy RESidual Sum and Outlier (MR-PRESSO) were employed. The MR-Egger method operates under the premise that an effect on the outcome persists even when the value of the exposure effect is zero [29]. By evaluating the MR-Egger intercept term against zero, a significant discrepancy suggests the presence of substantial horizontal pleiotropy [29]. The MR-PRESSO technique quantifies horizontal pleiotropy by adding the residuals for each SNP [30]. It includes a global test to determine the overall level of pleiotropy among IVs and an outlier test to identify specific SNPs that contribute anomalously to the detected pleiotropy [30]. Only findings that have passed the heterogeneity and horizontal pleiotropy tests will be included in this study. We further plotted leave-one-out analyses to assess whether our results were significantly influenced by any single SNP, thereby supporting the robustness of our findings. To avoid potential reverse causality, we conducted a reverse MR analysis. The methods and settings used were consistent with those in the discovery stage.

**Figure 1:**
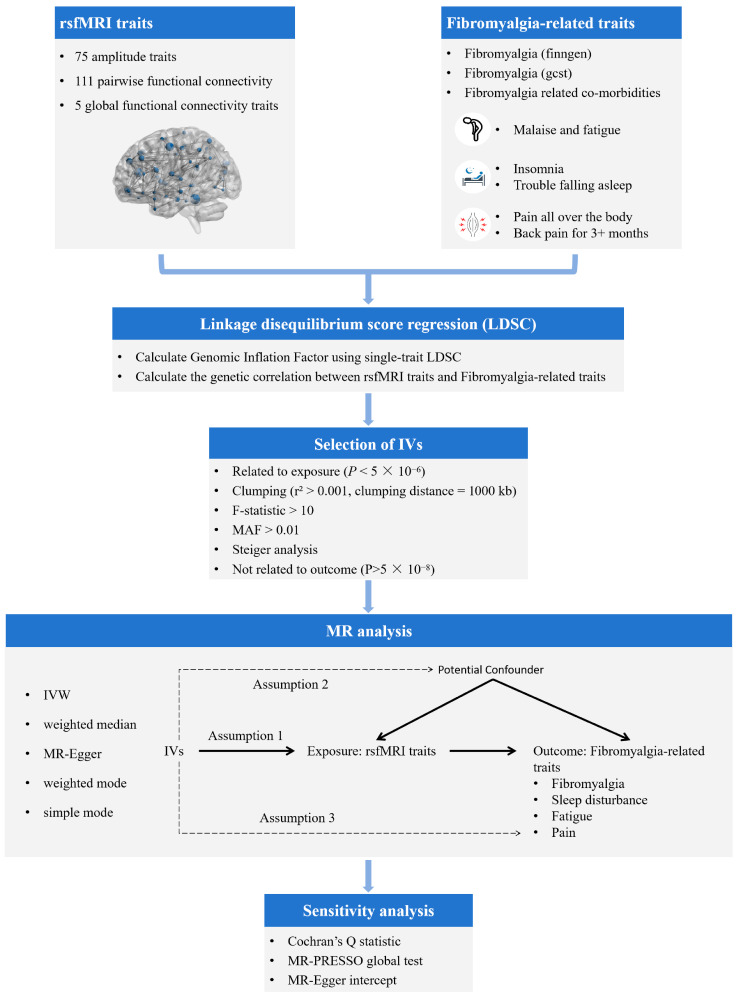
Workflow of two-sample mendelian randomization. Assumption 1: the IVs must be strongly associated with the exposures; Assumption 2: the IVs must be independent of the potential confounders of the association between the exposure and outcome; Assumption 3: the IVs should not be associated with the outcomes directly.

The complete analysis flow is shown in Figure 1. All Mendelian Randomization (MR) analyses were conducted in R version 4.3.1, available at https://www.r-project.org/ (accessed on 11 February 2025), utilizing the “TwoSampleMR”. *p* < 0.05 was considered as statistically significant in genetic correlation and MR analyses.

## 3. Results

### 3.1. LDSC Results

**Figure 2:**
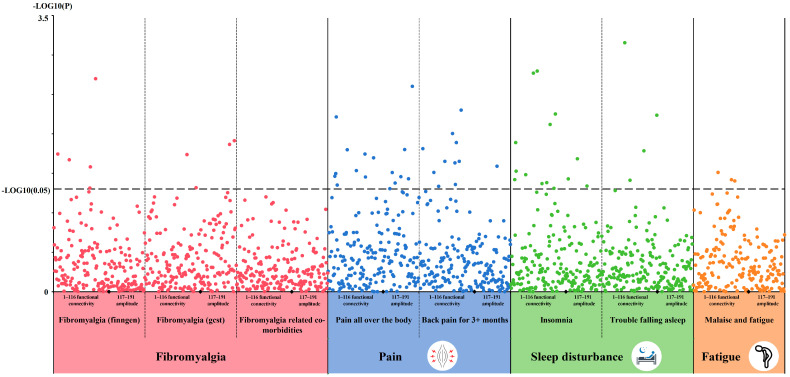
LDSC analysis of the genetic correlation between rsfMRI traits and fibromyalgia-related traits. Significant correlations were observed between 4 rsfMRI traits and “Trouble falling asleep”, 11 rsfMRI traits and “Back pain for 3+ months”, 16 rsfMRI traits and “Pain all over the body”, 14 rsfMRI traits and “Insomnia”, 5 rsfMRI traits and “Fibromyalgia (finngen)”, 4 rsfMRI traits and “Fibromyalgia (GCST)”, and 3 rsfMRI traits and “Malaise and fatigue”. *p* < 0.05 was considered significant.

We conducted single-trait LDSC analysis using GWAS summary data to detect inflation of statistical quantities of exposures and outcomes. The LDSC results indicated that all 191 rsfMRI traits exhibited heritability estimates greater than zero (Table S1). The highest observed heritability was 0.3628, with an average of 0.1159. Among the traits related to fibromyalgia, the heritability of “Back pain for 3+ months” was the highest at 0.0443, whereas “Fibromyalgia related co-morbidities” had the lowest heritability of 0.0032 (Table S2). The lambda GC values for the eight fibromyalgia-related phenotypes ranged from 1.0135 to 1.0895. We further explored the genetic correlations between 8 fibromyalgia-related phenotypes and the 191 rsfMRI traits using LDSC (Figure 2, Table S3). Significant correlations were observed between 4 rsfMRI traits and “Trouble falling asleep”, 11 rsfMRI traits and “Back pain for 3+ months”, 16 rsfMRI traits and “Pain all over the body”, 14 rsfMRI traits and “Insomnia”, 5 rsfMRI traits and “Fibromyalgia (finngen)”, 4 rsfMRI traits and “Fibromyalgia (gcst)”, and 3 rsfMRI traits and “Malaise and fatigue”. However, no significant correlations were observed with the rsfMRI traits for “Fibromyalgia related co-morbidities”.

### 3.2. Causal MR Associations Between rsfMRI Traits and Fibromyalgia-Related Traits

**Figure 3:**
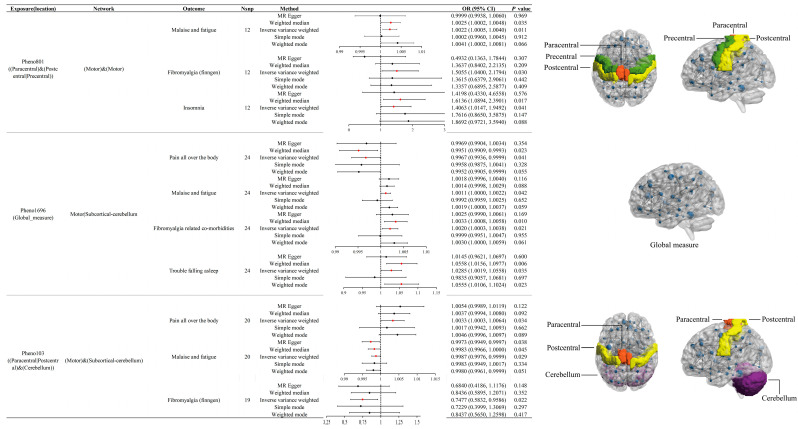
Mendelian randomization results of rsfMRI traits and fibromyalgia-related traits. **Left**: the forest plot shows the significant causal relationships estimated using five MR methods (inverse variance weighted, weighted mode, weighted median, simple mode, and MR Egger). The red dots represent rsfMRI traits that have a significant effect on fibromyalgia-related traits, while the black dots represent those with no significant effect. *p* < 0.05 was considered significant. OR represents the effect size of a 1 standard deviation change in the mean rsfMRI phenotype on the risk of fibromyalgia, with the error bars indicating 95% confidence intervals. *p*-values are from the Mendelian randomization analyses, and all analyses were two-sided. **Right**: the pattern diagram illustrates the brain anatomical region corresponding to the rsfMRI phenotypes.

In the discovery stage, we identified a total of 81 potential causal effects (Table S5), involving 61 rsfMRI traits and 8 fibromyalgia-related traits (Table S5). Notably, we found that Pheno801, Pheno1696, and Pheno103 may have significant causal effects on three or more fibromyalgia-related traits, including Fibromyalgia (finngen), fibromyalgia-related co-morbidities, insomnia, malaise and fatigue, pain all over the body, and trouble falling asleep (Figure 3, Table S6). Pheno801 is characterized by connectivity within the paracentral, postcentral, and precentral regions, reflecting a motor network primarily associated with sensorimotor functions. Pheno1696 represents a global measure implicating motor and subcortical-cerebellar networks, reflecting broader motor coordination. Pheno103 involves the paracentral and postcentral regions in conjunction with the cerebellum, highlighting a functional network linked to both motor and subcortical-cerebellar systems. All results were subjected to evaluations of pleiotropy and heterogeneity (Table S7).

### 3.3. Effects of Pheno801 on Fibromyalgia-Related Traits

The Pheno801 trait is related to the activity of the paracentral, postcentral, or precentral regions and is associated with the motor network. IVW results indicate that Pheno801 has significant causal effects on malaise and fatigue (OR = 1.0022, 95% CI: 1.0005 to 1.0040, *p* = 0.01), Fibromyalgia (finngen) (OR = 1.5055, 95% CI: 1.0400 to 2.1794, *p* = 0.03), and insomnia (OR = 1.4063, 95% CI: 1.0147 to 1.9492, *p* = 0.04). In the replication stage, its effect on insomnia was replicated (OR = 2.1190, 95% CI: 1.0560 to 4.2521, *p* = 0.03). Additionally, the direction of effect on Fibromyalgia (finngen) (OR = 1.2680, *p* = 0.62) and malaise and fatigue (OR = 1.0043, *p* = 0.05) remained consistent with the discovery stage.

### 3.4. Effects of Pheno1696 on Fibromyalgia-Related Traits

The Pheno1696 trait is associated with the motor or subcortical-cerebellum and represents a phenotype based on global measures. IVW results suggest that Pheno1696 acts as a risk factor for fibromyalgia-related comorbidities (OR = 1.002, 95% CI: 1.000 to 1.004, *p* = 0.02). It is also linked to increased risks of trouble falling asleep (OR = 1.0285, 95% CI: 1.003 to 1.0558, *p* = 0.04) and malaise and fatigue (OR = 1.0011, 95% CI: 1.0000 to 1.0022, *p* = 0.04), while providing a protective effect against pain all over the body (OR = 0.9967, 95% CI: 0.9936 to 0.9999, *p* = 0.04). These associations were further validated in the replication stage, with Pheno1696 continuing to show significant risk effects for trouble falling asleep (OR = 1.0595, 95% CI: 1.0124 to 1.1087, *p* = 0.01) and malaise and fatigue (OR = 1.0020, 95% CI: 1.0001 to 1.0038, *p* = 0.04). Furthermore, the directions of effect for Pheno1696 on fibromyalgia-related comorbidities (OR = 1.0030, *p* = 0.05) and pain all over the body (OR = 0.9972, *p* = 0.61) remained consistent with discovery stage.

### 3.5. Effects of Pheno103 on Fibromyalgia-Related Traits

The Pheno103 trait, associated with the activity of the paracentral or postcentral regions and the cerebellum, is linked to the motor and subcortical-cerebellum networks. According to IVW results, Pheno103 acts as a protective factor against Fibromyalgia (finngen) (OR = 0.7477, 95% CI: 0.5832 to 0.9586, *p* = 0.02) and malaise and fatigue (OR = 0.9987, 95% CI: 0.9976 to 0.9999, *p* = 0.03), but it is a risk factor for pain all over the body (OR = 1.0033, 95% CI: 1.003 to 1.0064, *p* = 0.03). In the replication stage, although no significant effects were observed, the results indicated the same direction of effect as in the discovery phase (Fibromyalgia (finngen): OR = 0.8712, *p* = 0.50; malaise and fatigue: OR = 0.9985, *p* = 0.11; pain all over the body: OR = 1.0050, *p* = 0.40).

### 3.6. Sensitivity Analysis

According to the results of the MR-Egger intercept and MR-PRESSO global tests, there was no evidence of horizontal pleiotropy (Table S7). In addition, no significant heterogeneity was found in the Cochran’s Q test (Table S7). The leave-one-out plots show that the trend of the results remains consistent after excluding any single SNP (Figure S1). No potential reverse causality was observed in the reverse MR analysis (Table S8).

## 4. Discussion

**Figure 4:**
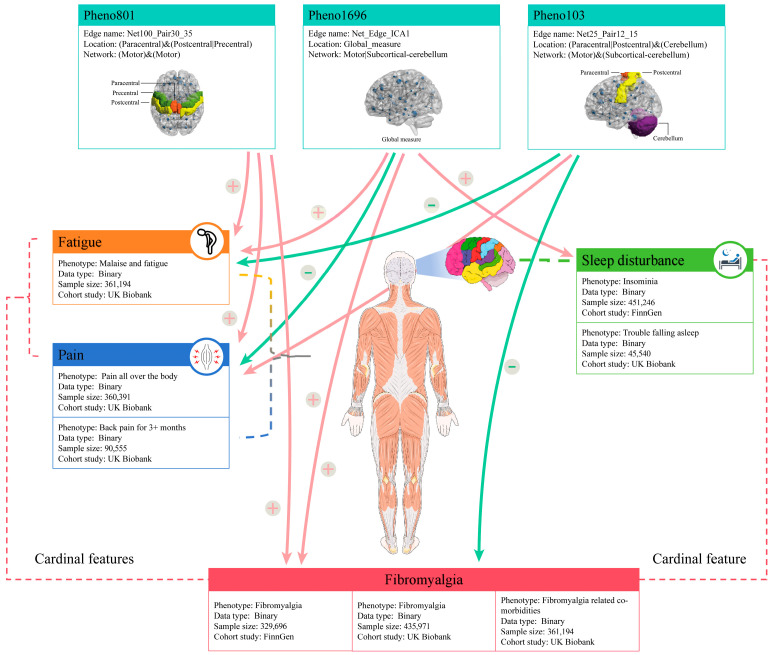
Interaction between rsfMRI traits and fibromyalgia-related traits. This figure highlights a total of ten causal effects involving three rsfMRI traits and six fibromyalgia-related traits. The red plus signs (+) and arrows signify risk factors, whereas the green minus signs (−) and arrows denote protective factors.

Fibromyalgia is a complex, chronic pain condition that primarily affects the musculoskeletal system, although it can involve other areas as well [1]. It is characterized by three cardinal features: idiopathic pain symptoms, fatigue, and sleep disturbances (Figure 4) [1,31]. Patients with fibromyalgia often use a variety of pain descriptors, and their pain is frequently likened to neuropathic pain [32,33]. Fatigue can be either physical or mental, with a wide range of severity from mild tiredness to a profound exhaustion akin to that experienced during viral infections such as influenza [1,34]. Sleep problems include various forms of insomnia and frequent awakenings. Non-restorative sleep is particularly prevalent; even when the quality and duration of sleep appear normal, individuals with fibromyalgia frequently report a lack of restorative rest [35].

The current focus of research is on the role of abnormal neurophysiological processes within the brain and spinal cord in the pathogenesis of fibromyalgia [36]. Substantial evidence suggests that central sensitization is a key pathophysiological mechanism in FM [37]. Central sensitization refers to the increased responsiveness of the central nervous system to various stimuli, such as pressure, temperature, light, and medication [38]. Napadow et al. found that fibromyalgia patients exhibited greater connectivity within the Default Mode Network (DMN) and the right Executive Attention Network (rEAN), with significant connectivity between the DMN and the insular cortex, a region associated with pain processing [11]. Hsiao et al. confirmed the frequency-specific reorganization of the insula–DMN connectivity in patients with fibromyalgia [39]. Cifre et al. reported a substantial imbalance in the connectivity of pain networks during rest among fibromyalgia patients [10]. Cagnie et al. showed that there is moderate evidence for significant imbalances in the resting-state functional connectivity among pain-processing regions and cognitive-attention networks of the brain in patients with fibromyalgia [40]. An important question that remains is whether certain individuals possess a higher inherited propensity for developing central sensitization and whether this increases their risk of developing pain hypersensitivity and its chronicity [37]. In this study, we conducted a two-sample Mendelian randomization analysis to investigate the causal relationships between 191 rsfMRI traits and fibromyalgia-related traits. The results revealed a causal link between brain resting-state functional networks and fibromyalgia, highlighting that Pheno801, Pheno1696, and Pheno103 may exert causal effects on multiple fibromyalgia-related traits, warranting broader attention to their roles in this condition. This study establishes a causal relationship between brain functional networks and fibromyalgia, offering valuable insights into the disease’s pathophysiology. Furthermore, it provides a basis for future interventions using repetitive transcranial magnetic stimulation (rTMS) or non-invasive brain stimulation (NIBS) targeting specific brain regions to normalize the hyperactivity of central nervous system functions, thereby contributing to the treatment of fibromyalgia.

The mechanisms of central sensitization in fibromyalgia patients can be discussed from two perspectives: diminished pain inhibition and enhanced pain perception. Research by Jensen and colleagues has shown that in the primary link of the descending pain regulating system, the rostral anterior cingulate cortex, fibromyalgia patients failed to respond to pain provocation [41,42]. Additionally, FM patients exhibited reduced connectivity within the brain’s pain inhibitory network during calibrated pressure pain compared to healthy controls [41]. Truini et al. discovered abnormal resting-state functional connectivity of the periaqueductal gray (PAG), which may lead to impaired descending pain inhibition [43]. Similarly, Vanneste et al. identified that the connectivity inhibition between the dorsal lateral prefrontal cortex and the posterior cingulate cortex on the pain inhibitory pathway was constrained by decreased functional connectivity with the pregenual anterior cingulate cortex [44]. Conversely, Giesecke et al. found that, at equal levels of pressure, patients with fibromyalgia experienced significantly more pain and showed more extensive, common patterns of neuronal activation in pain-related cortical areas [45]. Pujol et al. noted that, upon pain stimulation, both fibromyalgia patients and control groups had comprehensive activation of pain-related regions, but fibromyalgia patients exhibited significantly larger activation in the anterior insula–basal ganglia complex and the cingulate cortex [46]. Moreover, Burgmer and colleagues observed a unique temporal brain activation of the frontal cortex in patients with FM and noted a specific relationship between brain activity during pain anticipation and the magnitude of the subsequent pain experience in the motor cortex and the cingulate cortex [47]. Therefore, further studies into the central mechanisms, whether through interventions to enhance pain inhibition or to alleviate pain perception, may serve as potential therapeutic approaches to improve the quality of life for fibromyalgia patients.

Our research institute has identified three rsfMRI phenotypes that involve the motor network; additionally, Pheno1696 and Pheno103 also involve the subcortical-cerebellar network. Specifically, these phenotypes are associated with the paracentral, precentral, postcentral, and cerebellar regions. Cifre and colleagues have found that patients with fibromyalgia exhibit increased functional connectivity of the primary motor cortex with the supplementary motor area and decreased connectivity between the secondary somatosensory area and the motor cortex [10]. Markus’ studies indicate differences in the activation of the supplemental motor areas between fibromyalgia patients and controls [48]. Research by Schmidt-Wilcke shows that, compared to controls, fibromyalgia patients have increased gray matter in the left cerebellum [49]. Giesecke and colleagues have noted that, under similar stimulus intensities, the cerebellum activation is higher in fibromyalgia patients [45]. Lutz’s work has documented a decrease in gray matter volume and an increase in fractional anisotropy in the postcentral gyri of fibromyalgia patients [50]. Currently, there is no literature reporting abnormalities in the paracentral and precentral regions in fibromyalgia patients. However, considering the functional connections between different brain areas and their relationships with the motor network, these regions may still play a role in the development of fibromyalgia.

Changes in the structure and function of the motor cortex and the supplementary motor area may be one of the pathophysiological mechanisms of fibromyalgia [51,52,53]. For example, as measured using Functional Near-Infrared Spectroscopy (fNIRS) during a fast finger tapping task, individuals with fibromyalgia demonstrate an abnormal task-response with significantly lower oxyhemoglobin concentration in the motor cortex [54]. Additionally, hyperexcitability of motor networks occurs during acute painful stimulation in fibromyalgia patients, further suggesting dysfunctional motor cortex systems [55]. In a study by Park et al., it was found that the reward system of the motor network was dysregulated in fibromyalgia patients. These patients exhibited greater engagement of the motor network during both early and late gain anticipation phases, suggesting an overall enhancement of the motor network response during anticipation of gains/reward [56]. Furthermore, the cerebellum plays a role in pain perception, as manifested in the posterior lobe of the cerebellum, which is crucial for understanding the adaptability related to pain in motor control [57,58,59]. Research has shown that a lower grey matter volume in the cerebellum may reflect impairments in cognitive control, emotional processing, and pain perception in fibromyalgia [60,61]. Therefore, abnormalities in the motor network and cerebellar network function may play a significant role in the pathogenesis of fibromyalgia.

Although there is currently no existing intervention that directly targets the three functional connectivity networks we identified, existing findings demonstrate that appropriate interventions can alter the functional connectivity of certain regions within these networks. In Pineda-Pardo et al.’s study, it was found that, after transcranial magnetic stimulation (tSMS), the functional connectivity of the paracentral cluster, including the right superior temporal gyrus, postcentral gyrus, superior parietal lobe, and postcentral gyrus, significantly increased [62]. In Wei et al.’s study, it was observed that after low-frequency repetitive transcranial magnetic stimulation (rTMS), both the centrality of the left paracentral lobule (PCL) and its degree centrality significantly decreased, while the functional connectivity between the left primary motor cortex and the PCL significantly decreased. Additionally, high-frequency stimulation led to a decrease in the centrality of the right supplementary motor area (SMA). On the other hand, after high-frequency rTMS, functional connectivity between the right SMA and the right precentral gyrus significantly increased [63]. Yao et al.’s study found that bilateral cerebellar rTMS could also cause changes in cerebellar functional connectivity. Specifically, the connectivity between the left cerebellar Crus II and the right dorsolateral prefrontal cortex, bilateral medial prefrontal cortex, and bilateral cingulate cortex was enhanced. Furthermore, the connectivity between the right cerebellar Crus II and both the DLPFC and the medial prefrontal cortex was also increased [64]. Therefore, it is of great value to explore in the future the use of various intervention measures such as rTMS and non-invasive brain stimulation (NIBS) to adjust the brain functional network connections of patients with fibromyalgia, thereby improving the quality of life of patients with fibromyalgia.

Our research is the first to utilize Mendelian randomization to reveal potential causal effects between resting-state functional magnetic resonance imaging (rsfMRI) and fibromyalgia. Unlike traditional MRI, which reflects the structural characteristics of specific brain regions, rsfMRI directly measures the functional activity levels of these regions, thereby uncovering the intrinsic and stable networks of the human brain [65]. This offers valuable insights into the abnormal functional connectivity observed in fibromyalgia. Moreover, Mendelian randomization leverages genetic variants that are randomly distributed within a population and remain constant from birth, facilitating the establishment of causal relationships with modifiable risk factors [66,67]. This methodology effectively addresses the limitations of observational studies by controlling for measurable confounding factors [66,67]. The article still has some limitations: First, the rsfMRI data and certain fibromyalgia-related traits GWAS utilized in our study involve the UK Biobank cohort, which may lead to a degree of sample overlap. Secondly, all selected GWAS are sourced from European populations. Due to genetic differences, the genetic structure and disease-associated risk loci may exhibit heterogeneity in Asian, African, and other non-European populations. Thirdly, the exposure and partial outcome data used in our research all included data from the UK Biobank (UKB), thus resulting in some sample overlaps. Although we aimed to mitigate this limitation by including outcome data from an independent cohort (FinnGen), we acknowledge that the presence of overlapping samples could still affect the robustness of the causal inference. Additionally, while our current Mendelian randomization analysis has identified potential causal relationships between rsfMRI and fibromyalgia-related traits, given the methodological limitations, such as the validity of instrumental variables and residual confounding, our results should still be interpreted with caution. Therefore, further experimental or randomized controlled studies are needed to confirm the role of rsfMRI in the progression of fibromyalgia, providing stronger references for improving treatment outcomes.

## 5. Conclusions

The findings demonstrate a significant genetic correlation between brain functional networks and fibromyalgia. Notably, our research identified three rsfMRI traits that involve the motor network and the subcortical-cerebellar network, with the involved regions including the parafcentral region, precentral region, posterior central region, and cerebellar region. These phenotypes may have potential causal associations with various characteristics related to fibromyalgia. Therefore, these levels of brain network function may be potential research targets for the intervention and treatment of fibromyalgia.

## Data Availability

The datasets for the brain rsfMRI can be obtained via Zenodo at https://zenodo.org/records/5775047 (accessed on 18 August 2024). GWAS summary statistics for Fibromyalgia (FinnGen) and insomnia were obtained from the FinnGen R11 release and can be accessed in accordance with the guidelines provided on their website (https://www.finngen.fi/en/access_results, accessed on 18 August 2024). GWAS data on pain all over the body, back pain for 3+ months, malaise and fatigue, fibromyalgia-related comorbidities, and trouble falling asleep was obtained from the Neale Lab (http://www.nealelab.is/uk-biobank, accessed on 18 August 2024). GWAS data for Fibromyalgia (gcst) were downloaded from the NHGRI-EBI GWAS Catalog for study GCST90129439 (https://www.ebi.ac.uk/gwas/home, accessed on 18 August 2024).

## Supplementary Materials

Supplementary materials can be downloaded at Table S1–S8 and Figure S1 can be downloaded at https://zenodo.org/records/15715844 (accessed on 20 June 2025). Figure S1: The leave-one-out plots of Pheno801, Pheno1696, and Pheno103; Table S1. Description of single trait LDSC test results of 191 rsfMRI traits; Table S2. Single trait LDSC test results of 8 Fibromyalgia-related traits; Table S3. Pairwise LDSC analysis results of rsfMRI traits and Fibromyalgia-related traits; Table S4. IVs used in the MR analysis; Table S5. Significant results of MR; Table S6. Significant MR results of Pheno801, Pheno1696, and Pheno103; Table S7. Results of heterogeneity and horizontal Pleiotropy tests; Table S8. Results of reverse MR.
